# Supplementary material for: Vaccine Quality Is a Key Factor to Determine Thermal Stability of Commercial Newcastle Disease (ND)Vaccines
Source: Vaccines (Basel). 2021 Apr 9;9(4):363. doi: 10.3390/vaccines9040363 (PMC8069011; doi:10.3390/vaccines9040363)
Supplement: Supplementary file 1 [file vaccines-09-00363-s001.pdf]

Supplemental Table S1: Stability of HAU in lyophilized ND-vaccines (A-F)

Table S1c: Lyophilized ND-vaccines (A-F) at 4 °C.

	HA-Titer (log2)					
**Time (days)**	**A**	**B**	**C**	**D**	**E**	**F**
0	7	7	9	8	9	6
7	7	8	9	7	9	6
21	7	8	9	7	9	6

Table S1b: Lyophilized ND-vaccine (A-F) at 37 °C.

	HA-titer (log2)					
**Time (days)**	**A**	**B**	**C**	**D**	**E**	**F**
0	7	7	9	8	9	6
1	7	7	9	8	8	6
3	7	7	9	8	8	6
5	7	7	9	8	8	6
7	7	7	9	8	8	6
10	7	7	9	8	9	6
14	7	7	9	8	9	6
21	7	8	9	8	9	6

Table S1c: Dissolved ND-vaccines (A-F) at 37 °C.

	HA-Titer (log2)					
**Time (days)**	**A**	**B**	**C**	**D**	**E**	**F**
0	7	9	9	6	9	6
0,5	8	9	9	6	8	6
1	7	10	9	6	8	6
3	7	7	9	7	9	6
4	7	9	9	7	8	6

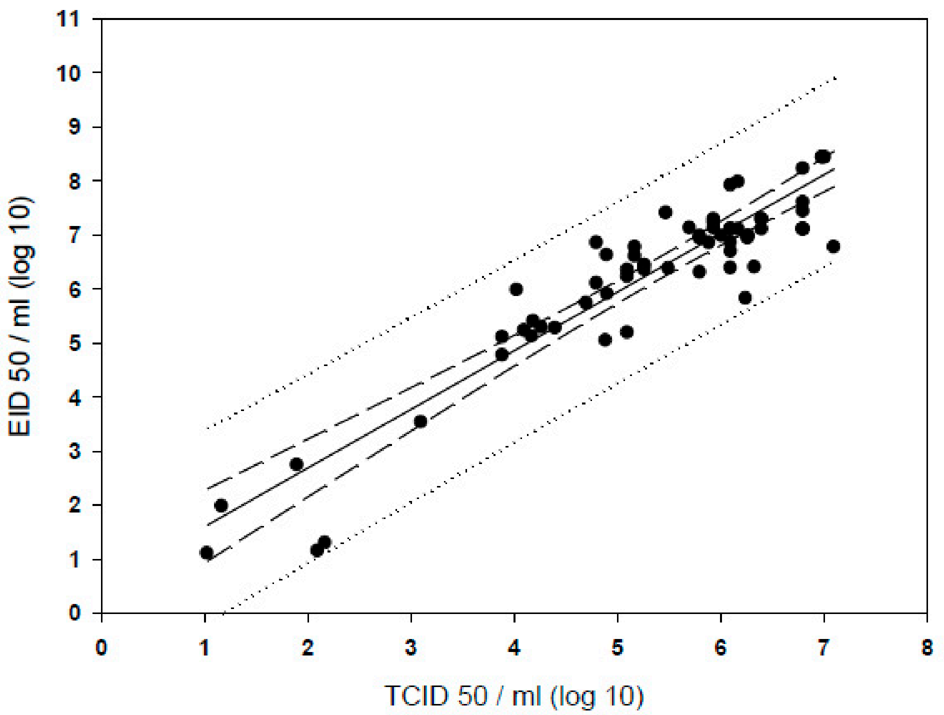

Figure S1: Correlation of titer obtained by egg or cell culture. Supernatants of all lentogenic virus stocks (n= 67) were titrated in parallel in SPF embryonated eggs as well as on LMH cells. Depicted is the relation between titer as egg infectious doses (EID50) and tissue culture doses (TCID50) with the correlation as well as the 95% Confidence interval (dashed lines) and the prediction intervals (dotted lines).
